# The correlation between serum calcium and asprosin in elderly patients with type 2 diabetes mellitus in community

**DOI:** 10.3389/fendo.2026.1752403

**Published:** 2026-06-09

**Authors:** Dong Liang, Huimin He, Zhengqian Wang, Chunfan Niu, Yan Wang, Linxin Xu, Jing Yang

**Affiliations:** 1 Department of Endocrinology, First Hospital of Shanxi Medical University, Shanxi Medical University, Taiyuan, Shanxi, China; 2 First Clinical Medical College, Shanxi Medical University, Taiyuan, Shanxi, China; 3 Shanxi Innovation Center for Integrated Management of Hypertension, Hyperlipidemia and Hyperglycemia Correlated with Cardiovascular and Cerebrovascular Diseases, Taiyuan, China; 4 Clinical Research Center for Endocrine and Metabolic Diseases of Shanxi Medical University, Taiyuan, China

**Keywords:** asprosin, calcium, CaSR, elderly, type2 diabetes mellitus

## Abstract

**Objective:**

This study aims to investigate the association between serum calcium and asprosin levels in community-dwelling elderly patients with type 2 diabetes mellitus (T2D). It seeks to clarify the relationship between serum calcium and other metabolic indicators, as well as identify independent factors influencing serum calcium levels. The findings are intended to provide a foundation for elucidating the interactive mechanisms between calcium metabolism and adipokines.

**Methods:**

A total of 321 elderly T2D patients aged ≥65 years were enrolled from November 2019 to July 2021 at the Zhuoma Community Health Service Station and Chengbei Xijie Community Health Service Center in Changzhi City, Shanxi Province. General information including age, duration of diabetes, body mass index (BMI), etc., was collected. Fasting plasma glucose (FPG), glycated hemoglobin A1c (HbA1c), creatinine (CRE), high-density lipoprotein cholesterol (HDL-C), uric acid (UA), and other biochemical indicators were measured. Serum asprosin levels were determined using enzyme-linked immunosorbent assay (ELISA) while serum calcium levels were assessed with an automatic biochemical analyzer. Pearson/Spearman correlation analysis was employed to evaluate associations between serum calcium and various indicators; multiple linear regression analysis was utilized to identify independent factors affecting serum calcium levels.

**Results:**

Serum asprosin levels increased progressively across serum calcium tertiles. Correlation analyses demonstrated statistically significant associations between serum calcium and HDL-C (r = 0.123), uric acid (r = 0.132), asprosin (r = 0.124), and creatinine (r = −0.113). In multivariable linear regression analysis, creatinine (standardized β = −0.179, p = 0.003) and asprosin (standardized β = 0.187, p = 0.002) remained independently associated with serum calcium levels. Pearson correlation analysis demonstrated a significant positive association between serum asprosin and total calcium (r = 0.214, P < 0.001). A similar positive correlation was observed between asprosin and albumin-corrected calcium (r = 0.170, P = 0.002).

**Conclusion:**

Serum asprosin was independently associated with serum calcium levels in elderly patients with T2D, suggesting a potential link between calcium homeostasis and adipokine regulation. Further longitudinal and mechanistic studies may help elucidate the clinical and biological implications of this relationship.

## 1 Introduction

The accelerated global aging process has led to a continuous increase in the number of elderly individuals with type 2 diabetes mellitus (T2D). Currently, China has approximately 47.9 million elderly diabetes patients, the largest such population in the world. The accompanying metabolic disorders and cardiovascular complications have become significant public health challenges (1). Owing to age-related physiological decline, a high prevalence of comorbidities and complex medication regimens, the interactions among metabolic parameters in the elderly have become increasingly complex. Dysregulated calcium homeostasis and adipokine metabolism have been confirmed as key factors in the pathological progression of T2D and the development of its complications (2, 3).

Serum calcium, a critical electrolyte necessary for maintaining normal physiological functions, plays a pivotal role in regulating insulin secretion (4). Existing research indicates that disturbances in calcium homeostasis can contribute to the pathogenesis of T2D (5, 6). Moreover, several large-scale prospective cohort studies have found a significant association between abnormal serum calcium levels and increased risk of cardiovascular disease and all-cause mortality in the elderly population (7–16). These findings suggest that serum calcium may serve as a potential indicator for assessing metabolic and cardiovascular risk in elderly T2D patients.

Asprosin, a fasting-induced glucogenic hormone secreted by white adipose tissue, was first identified by Romere et al. (17). In their seminal work, asprosin was shown to stimulate hepatic glucose production and regulate appetite via hypothalamic neurons, establishing it as a key mediator of energy homeostasis (17). Subsequent studies have demonstrated that circulating asprosin levels are elevated in metabolic disorders, including obesity and type 2 diabetes, implicating it in systemic glucose dysregulation (18, 19). Moreover, asprosin has been associated with polycystic ovary syndrome and insulin resistance, further highlighting its relevance in metabolic pathophysiology (20, 21). Emerging evidence also links elevated asprosin levels to cardiovascular complications such as myocardial injury, heart failure, and early-stage diabetic cardiomyopathy (22–24). Asprosin, like other adipokines, plays an important role in regulating metabolic functions beyond glucose homeostasis. Previous studies have mainly focused on how adipokines influence bone metabolism by regulating calcium and phosphorus excretion (25). However, research on the relationship between serum calcium and asprosin remains limited, and there is currently no clear clinical evidence supporting their combined effects on metabolic outcomes.

This study aimed to investigate the relationship between serum calcium levels and asprosin in 321 community-dwelling elderly T2D patients. It also analyzed the correlations between serum calcium and other metabolic markers and identified independent factors influencing serum calcium levels.

## 2 Materials and methods

### 2.1 Study participants

Elderly patients with Type 2 Diabetes Mellitus (T2D), aged 65 years and older, who sought medical attention at the Zhuoma Community Health Service Station and Chengbei Xijie Community Health Service Center in Changzhi City, Shanxi Province, from November 2019 to July 2021, were enrolled in this study. Inclusion criteria: (1) Diagnosis of T2D based on the World Health Organization’s criteria established in 1999 (26); (2) Age ≥65 years; (3) Availability of complete clinical data and willingness to participate in the study. Exclusion criteria: (1) Patients presenting with diabetic ketoacidosis or hyperosmolar hyperglycemic state; (2) Patients exhibiting severe organ dysfunction (e.g., liver or kidney failure), significant infections, or malignancies; (3) Patients facing communication barriers due to mental illness; (4) Patients unable to comply with study protocols;(5)Patients with hypoproteinemia(blood albumin<40g/L). The study received approval from the Ethics Committee of the First Affiliated Hospital of Shanxi Medical University (Approval No. 2019), and all participants provided informed consent prior to enrollment.

### 2.2 Data collection

Upon admission, patients’ height and weight were measured for calculating body mass index (BMI). Waist circumference and hip circumference were recorded for determining the waist-to-hip ratio (WHR). Additional general information collected included age, duration of diabetes, systolic blood pressure (SBP), and diastolic blood pressure (DBP). On the following morning, fasting venous blood samples were obtained for measuring fasting plasma glucose (FPG), fasting C-peptide levels (FC-P), creatinine concentration (CRE), total cholesterol levels (TC), triglycerides levels(TG), high-density lipoprotein cholesterol concentrations(HDL-C), low-density lipoprotein cholesterol concentrations(LDL-C)—all assessed using a Beckman automatic biochemical analyzer model BK-200 from USA—as well as uric acid levels(UA), aspartate aminotransferase activity(AST), alanine aminotransferase activity(ALT), and serum calcium levels. Glycated hemoglobin A1c(HbA1c) was quantified via high-performance liquid chromatography using Roche model 501 from Switzerland.

### 2.3 Serum asprosin measurement

Patients underwent an 8–10 hour fasting period prior to blood collection in the morning under fasting conditions. Peripheral venous blood was drawn and subsequently centrifuged at 3000 rpm with a centrifugal radius of 13.5 cm for 15 minutes; the supernatant was then stored at -80 °C. Serum asprosin concentrations were measured using a commercially availableenzyme-linked immunosorbent assay (ELISA) kits according to the manufacturer’s instructions. Prior to analysis, all serum samples were randomly assigned for ELISA measurement to minimize potential batch-related bias. Each sample was assayed in triplicate, and the mean of the three measurements was used for the final analysis. The intra- and inter-assay coefficients of variation reported in this study were based on the manufacturer’s specifications.

### 2.4 Statistical analysis

Statistical analyses were conducted utilizing SPSS version 19.0 software. Normally distributed continuous variables are presented as mean ± standard deviation (x ± s) and compared using Student’s t-test. Non-normally distributed continuous variables are expressed as median (Q1, Q3) and assessed via the Mann-Whitney U test. Categorical variables are reported as counts (percentages) and compared using the χ² test. Pearson and Spearman correlation analyses were employed to evaluate correlations between serum asprosin levels and other clinical data. Additionally, multiple stepwise logistic regression analysis was performed to identify factors influencing serum calcium levels. A p-value of less than 0.05 was deemed statistically significant.

## 3 Results

### 3.1 Comparison of general characteristics, biochemical indicators, and asprosin in different calcium groups

A total of 321 elderly patients with T2D were included in the study, with a mean age of 71.85 ± 4.80 years; among them, 46.7% (150/321) were male. The median duration of diabetes was found to be 15 years (interquartile range: 10-20), and the average HbA1c level was recorded at 8.77 ± 1.78%. The results demonstrated that serum asprosin levels increased concomitantly with elevated serum calcium levels, and this difference was statistically significant (**Table 1**).

**Table 1:** Characteristics of patients with type 2 diabetes stratified by serum calcium tertiles.

Variable	T1 (<2.11 mmol/L) (n=101)	T2 (2.11–2.22 mmol/L) (n=120)	T3 (>2.22 mmol/L) (n=100)	*χ*^2^/*Z*/t	*P* for trend
Age (years)	72.04±4.57	72.18±4.98	72.21±5.18	0.035	0.966
SBP (mmHg)	133.82±19.88	133.41±15.90	137.69±19.94	1.696	0.185
DBP (mmHg)	73.85±10.11	75.41±10.50	75.43±9.14	0.847	0.430
WHR	0.943±0.077	0.937±0.079	0.939±0.078	0.161	0.851
FPG (mmol/L)	8.05±3.11	8.19±2.57	7.82±2.76	0.474	0.623
F-CP (ng/mL)	2.06±1.27	2.22±1.50	2.02±1.12	0.748	0.474
TC (mmol/L)	4.26±1.15	4.35±0.97	4.43±1.07	0.684	0.505
TG (mmol/L)	1.41(1.06, 1.96)	1.46(1.08, 2.11)	1.38(1.06, 2.06)	0.163	0.850
LDL-C (mmol/L)	2.50±0.86	2.59±0.81	2.52±0.86	0.354	0.702
HDL-C (mmol/L)	0.97±0.25	1.00±0.27	1.05±0.28	2.027	0.133
ALT (U/L)	19.39±21.30	17.88±10.74	19.56±11.85	0.418	0.659
AST (U/L)	19.98±14.00	18.77±7.03	21.49±9.50	1.861	0.157
GGT (U/L)	33.96±56.05	27.96±21.88	29.69±26.73	0.728	0.484
CRE (µmol/L)	77.89±38.40	71.57±22.85	73.38±23.28	1.355	0.260
HbA1c (%)	8.96±1.89	8.78±1.70	8.67±1.69	0.667	0.514
Asprosin (pg/mL)	356.18±107.07	363.68±113.70	394.09±103.18	3.464	0.032*
ALB (g/L)	49.3±3.01	49.4±2.88	49.8±2.79	0.990	0.373
UA (µmol/L)	329.34±93.73	351.28±119.31	366.14±113.04	2.824	0.061
Duration (years)	15.0(10.00, 20.00)	15.00(10.00, 20.00)	15.00(10.00, 20.00)	0.230	0.795
BMI (kg/m²)	26.07±3.31	25.05±3.72	25.12±3.38	2.537	0.081

SBP, Systolic Blood Pressure; DBP, Diastolic Blood Pressure; WHR, Waist-to-Hip ratio; FPG, Fasting Blood Glucose; F-CP, Fasting C-Peptide; TC, Total Cholesterol; TG,Triglycerides; LDL-C, Low-Density Lipoprotein cholesterol; HDL-C, High-Density Lipoprotein Cholesterol; ALT, Alanine Aminotransferase; AST, Aspartate Aminotransferase; GGT, Gamma-Glutamyl Transferase; CRE, Creatinine; HbA1c, Glycosylated Haemoglobin;ALB, Albumin; UA, Uric Acid; BMI, Body Mass Index .

Serum calcium Tertile 1<2.11mmol/L, Serum calcium Tertile 2 2.11-2.22mmol/L, Serum calcium Tertile 3 >2.22mmol/L.

*means P < 0.05.

### 3.2 Correlation between serum calcium and general characteristics, biochemical indicators, and asprosin

Pearson and Spearman correlation analyses indicated that serum calcium exhibited a positive correlation with high-density lipoprotein cholesterol (HDL-C), uric acid (UA), and asprosin while showing a negative correlation with creatinine (CRE). Multiple linear regression analysis further revealed that the CRE levels, and asprosin concentrations were independently associated with serum calcium levels. Specifically, asprosin showed positive correlations with serum calcium; conversely, CRE displayed a negative correlation with serum calcium—all differences being statistically significant (**Table 2**).

**Table 2:** Association between serum calcium and clinical variables in patients with type 2 diabetes.

Variable	Pearson correlation		Multiple linear regression	
	r	*P* for trend	Standardized β	*P* for trend
TC	0.078	0.164	–	–
LDL-C	0.039	0.487	–	–
HDL-C	0.123	0.029*		
SBP	0.050	0.374	–	–
DBP	0.068	0.228	–	–
WHR	-0.061	0.286	–	–
FPG	0.004	0.937	–	–
BMI	-0.073	0.194	–	–
F-CP	-0.031	0.582	–	–
ALT	0.003	0.957	–	–
AST	0.057	0.307	–	–
UA	0.132	0.018*		
CRE	-0.113	0.043*	-0.179	0.003
TG	-0.009	0.874	-0.009	0.874
Duration	-0.038	0.503	-0.038	0.503
Asprosin	0.214	<0.001*	0.187	0.002

*Spearman correlation analysis was used for skewness distribution.

SBP, Systolic Blood Pressure; DBP, Diastolic Blood Pressure; WHR, Waist-to-Hip ratio; FPG, Fasting Blood Glucose; F-CP, Fasting C-Peptide; TC, Total Cholesterol; TG,Triglycerides; LDL-C, Low-Density Lipoprotein cholesterol; HDL-C, High-Density Lipoprotein Cholesterol; ALT, Alanine Aminotransferase; AST, Aspartate Aminotransferase; GGT, Gamma-Glutamyl Transferase; CRE, Creatinine; HbA1c, Glycosylated Haemoglobin; UA, Uric Acid; BMI, Body Mass Index .

*means P < 0.05.

### 3.3 Multiple stepwise regression analysis of factors affecting serum calcium levels

In patients with type 2 diabetes, serum calcium levels were significantly associated with creatinine (CRE), albumin (ALB), and asprosin. Specifically, total serum calcium was negatively associated with CRE (β = –0.174, P = 0.002) and positively associated with ALB (β = 0.112, P = 0.039) and asprosin (β = 0.246, P < 0.001) (**Table 3**). Similarly, albumin-corrected calcium levels were negatively associated with CRE (β = –0.166, P = 0.002) and ALB (β = –0.330, P < 0.001), while remaining positively associated with asprosin (β = 0.235, P < 0.001) (**Table 4**).

**Table 3:** Correlation of serum calcium levels with CRE and asprosin in patients with type 2 diabetes.

Models	β coefficient	95% confidence interval	P value
	1.876	(1.643, 2.109)	<0.001
CRE µmol/L	-0.174	(-0.001, 0.000)	0.002
ALB g/L	0.112	(0.000-0.010)	0.039
asprosin pg/mL	0.246	(0.000, 0.000)	<0.001

CRE, Creatinine; ALB, Albumin.

**Table 4:** Correlation of albumin-corrected calcium levels with CRE and asprosin in patients with type 2 diabetes.

Models	β coefficient	95% confidence interval	P value
	2.676	(2.443, 2.909)	<0.001
CRE µmol/L	-0.166	(-0.001, 0.000)	0.002
ALB g/L	-0.330	(-0.020--0.010)	<0.001
asprosin pg/mL	0.235	(0.000, 0.000)	<0.001

CRE, Creatinine; ALB, Albumin.

### 3.4 Correlation between circulating asprosin and serum calcium parameters in patients with type 2 diabetes

**Figure 1:**
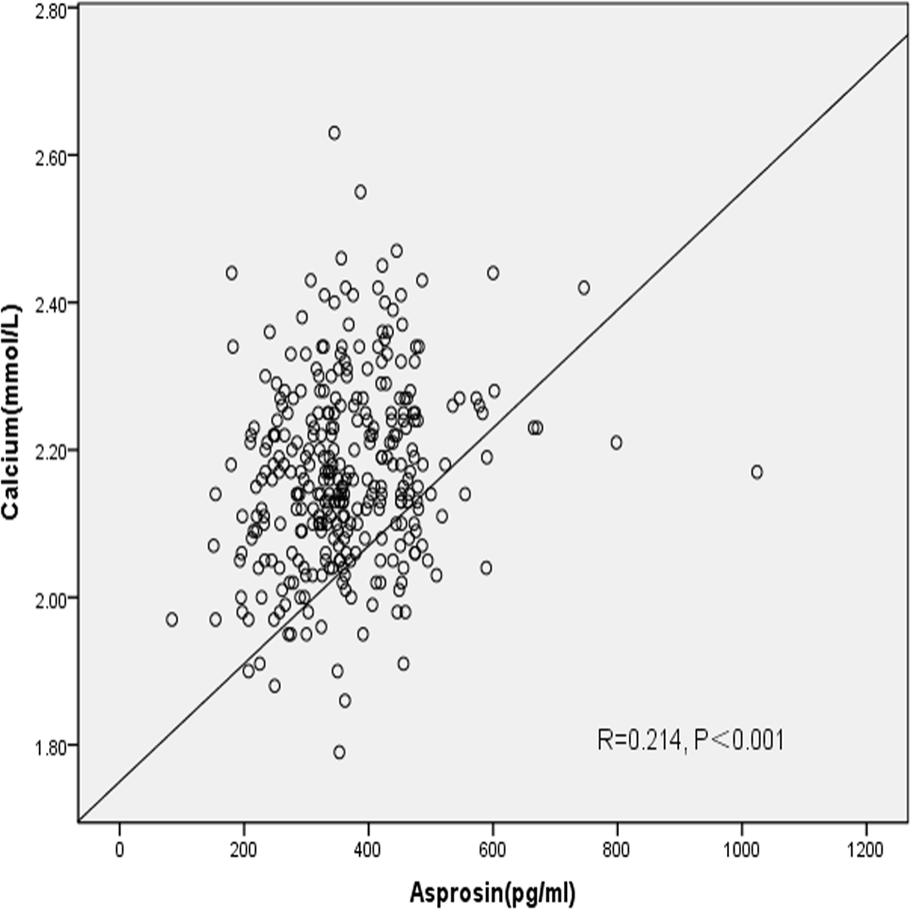
Association of serum calcium with Serum Asprosin in elderly Patients with Type 2 Diabetes mellitus.

**Figure 2:**
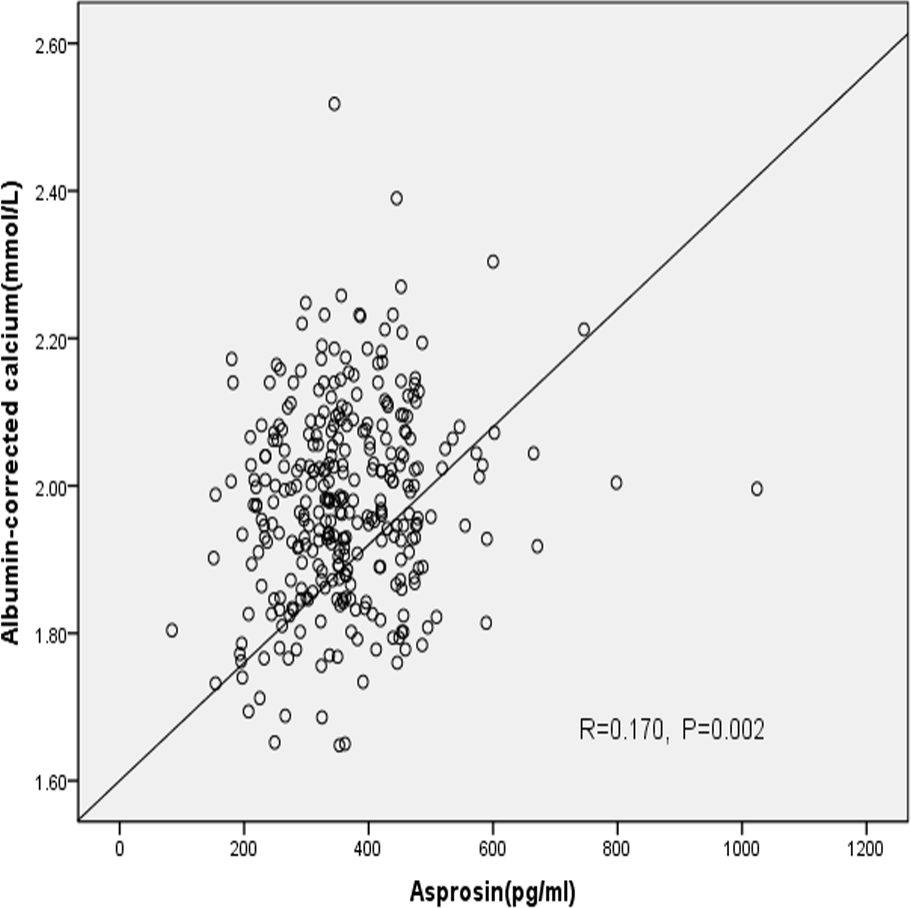
Correlation between circulating Asprosin and albumin-corrected serum calcium in elderly T2DM patients.

Scatter plot showing the Pearson correlation between asprosin and total serum calcium (r = 0.214, P < 0.001) (**Figure 1**). Scatter plot showing the Pearson correlation between asprosin and albumin-corrected calcium (r = 0.170, P = 0.002) (**Figure 2**).

### 3.5 Relationship between serum calcium and serum asprosin in elderly T2DM patients

**Figure 3:**
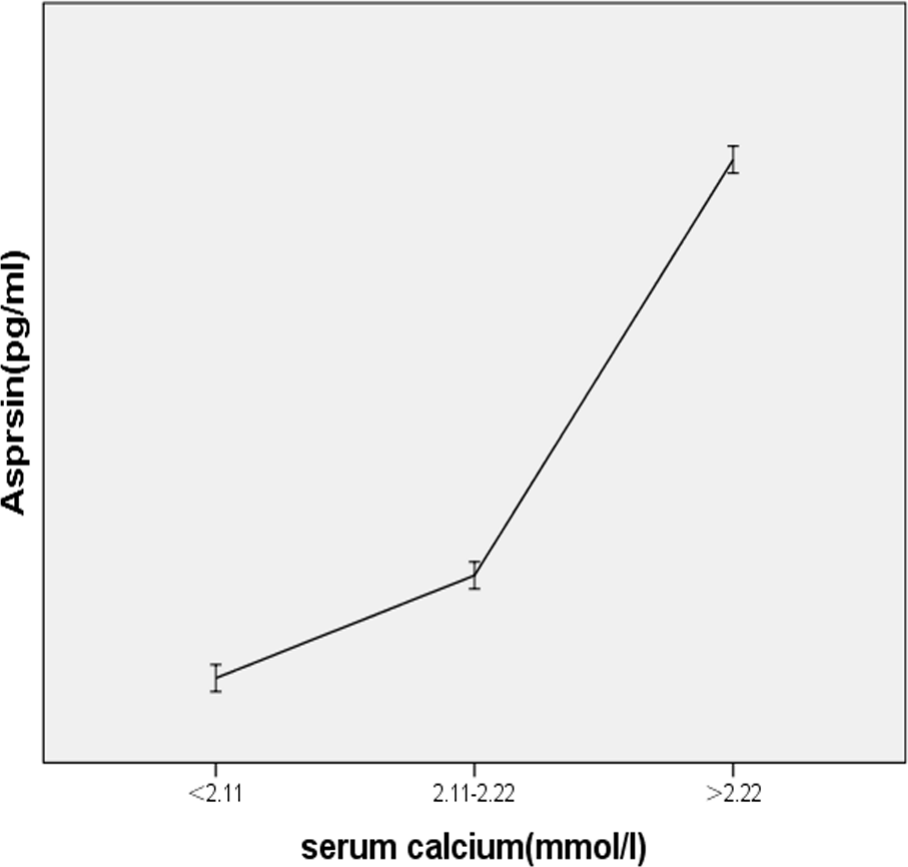
Relationship between serum calcium and serum asprosin in elderly T2DM patients.

All patients were categorized into three quantiles based on their serum calcium levels (T1 < 2.11 nmol/L, T2 2.11-2.22 nmol/L, T3 > 2.22 nmol/L). A general linear regression model was employed with serum asprosin as the dependent variable. The average serum asprosin levels (95% confidence interval) for the T1, T2, and T3 groups were found to be 356.18 (335.17, 377.73), 363.68 (344.97, 383.51), and 394.09 (373.13, 415.46), respectively. Compared to the T1 group, serum asprosin levels were significantly elevated in both of the other groups (P < 0.01). Notably, as serum calcium levels increased from T1 to T3, there was a corresponding gradual increase in average serum asprosin levels (**Figure 3**).

## 4 Discussion

The study revealed that serum asprosin levels progressively increased with rising serum calcium levels, demonstrating statistically significant differences. This finding suggests a potential link between these two biomarkers, which may be crucial for understanding metabolic disorders.

Asprosin, a fasting-induced glucogenic hormone secreted by white adipose tissue, has emerged as a key regulator of energy homeostasis, influencing important physiological processes such as appetite, hepatic glucose release, and insulin secretion (27–29). Elevated levels of asprosin are commonly observed in metabolic disorders, including obesity and type 2 diabetes (T2D), underscoring its role in glucose dysregulation and metabolic dysfunction (17–19). These findings align with prior research demonstrating that asprosin levels are significantly elevated in individuals with insulin resistance, polycystic ovary syndrome, and other metabolic conditions (20, 21, 30–33). Furthermore, asprosin has been implicated in cardiovascular complications, including myocardial injury, heart failure, and early-stage diabetic cardiomyopathy, highlighting its potential as a biomarker for cardiovascular dysfunction in T2D patients (22–24, 34).

In our study, we found that serum asprosin levels were significantly higher in elderly T2D patients with early cardiac dysfunction, suggesting that asprosin may serve as an early predictor of cardiac complications in this population (24). This observation is consistent with previous reports that link elevated asprosin levels with various aspects of cardiovascular disease (22, 23, 35). Additionally, exogenous asprosin has been shown to exert protective effects against diabetic cardiomyopathy by inhibiting autophagy, providing further evidence of its potential therapeutic role in managing T2D-related complications (36).

Serum calcium is a vital electrolyte within the human body and is essential for insulin secretion, myocardial/skeletal muscle contraction, and maintaining nerve function (37). Disruption of calcium homeostasis can lead to impaired insulin secretion due to interference with βcell calcium signaling. Moreover, it is associated with an increased risk of T2D (5, 6, 38) and heightened cardiovascular mortality along with all-cause mortality risks (7, 11). Previous studies have primarily concentrated on the relationship between asprosin and other metabolic parameters; however, no investigations have explored the association between asprosin and blood calcium—a commonly utilized clinical marker. Understanding their correlation could pave the way for more targeted therapeutic strategies for managing metabolic disorders.

Previous experimental studies have suggested complex interactions between adipokines and calcium signaling pathways. The calcium-sensing receptor (CaSR), a Class C G protein-coupled receptor widely expressed in adipocytes and other metabolic tissues, has been shown to participate in adipocyte differentiation, lipolysis regulation, and inflammatory responses (39–43). Moreover, several adipokines, such as leptin and adiponectin, are known to interact with calcium-dependent signaling pathways. Specifically, leptin has been shown to exert regulatory effects via the CaSR pathway, with its release being dependent on calcium ions (44, 45), while adiponectin modulates calcium and phosphorus balance through the Klotho pathway (26). Recent research suggests that asprosin may play a role through calcium in various organs. For instance, in the central nervous system, asprosin activates Agouti-related peptide Arcuate nucleus of the hypothalamus (AgRPARH) neurons via the Protein Tyrosine Phosphatase Receptor Type D (Ptprd) receptor—a process that depends on small conductance calcium-activated potassium (SK) channels (46). In skeletal muscle, treatment with asprosin has been demonstrated to inhibit messenger RNA expression of sarcoplasmic reticulum Ca2+/ATPase 2b (47). Given that both asprosin and serum calcium are commonly used indicators of T2D and its complications, elucidating the connection between these two indicators could aid in identifying high-risk groups and developing combined biomarkers.

However, there has yet to be a study investigating the relationship between blood calcium levels and asprosin in patients with T2D. Given that most adipokines closely interact with calcium, and considering that both asprosin and leptin are secreted by white adipose tissue while regulating appetite through modulation of agouti-related protein (AgRP), it is biologically plausible that asprosin and calcium metabolism may share common regulatory pathways, potentially involving CaSR-related signaling. Further mechanistic studies, including cellular and longitudinal investigations, are required to clarify whether calcium signaling directly influences asprosin secretion or whether the observed association reflects broader metabolic regulatory processes.

The positive association between asprosin and serum calcium was observed for both total calcium and albumin-corrected calcium were observed in this study. Although the strength of the correlations was modest, the direction and statistical significance of the relationship remained consistent after albumin correction. Given that total serum calcium is partially influenced by albumin concentration, the persistence of this association suggests that the observed relationship is not solely attributable to albumin-bound calcium, but may reflect a stable link between calcium homeostasis and adipokine regulation. Furthermore, it is plausible that asprosin influences calcium levels indirectly by modulating inflammation and metabolic parameters critical for maintaining calcium homeostasis. This research also elucidated the association between serum calcium and various indicators. Firstly, we identified a positive correlation between serum calcium and HDL-C, which is consistent with previous studies (48, 49). A cross-sectional study indicated that dietary calcium intake was positively associated with HDL-C in individuals with T2D (50). This relationship may be attributed to calcium signaling pathways involved in the PI3K/Akt/NF-κB and Nrf2/HO-1 pathways (51). However, some studies have reported that elevated serum calcium levels are negatively correlated with HDL-C, particularly among postmenopausal women (52). The discrepancy in findings may stem from differences in study populations. Additionally, our research revealed a positive correlation between serum calcium and UA, suggesting an interaction between uric acid metabolism and calcium homeostasis. The specific mechanisms underlying this relationship warrant further investigation; however, recent studies confirm that both serum calcium and uric acid can serve as independent predictors of cardiovascular risk (53). We also found that serum calcium was independently negatively correlated with serum creatinine. The kidneys play a crucial role in maintaining systemic calcium balance, and prior research has predominantly focused on patients with chronic kidney disease who exhibit lower serum calcium levels due to reduced renal excretion of this mineral and decreased intestinal absorption of it (37). Recent investigations indicate a positive correlation between elevated calcium levels and improved renal function (54).

Nevertheless, within our study population characterized by normal renal function, no similar associations have been documented. The independent association between creatinine and serum calcium suggests that renal handling of calcium may partially contribute to the observed relationship, as the kidney plays a central role in calcium homeostasis among elderly patients with T2D exhibiting normal renal function. Our study did not observe a correlation between serum calcium and blood glucose, HbA1c, or fasting C-peptide levels. These findings are inconsistent with those reported by Suh S et al., who identified a positive correlation between serum calcium and both fasting glucose and HbA1c (55, 56). However, they are partially consistent with the results of Dos Santos et al., who found a negative correlation between serum calcium and insulin levels (57). In addition, although HbA1c was not significantly associated with serum calcium in the present study, chronic hyperglycemia may indirectly influence mineral metabolism through inflammatory or renal mechanisms.Other reasons for these discrepancies may be attributed to variations in study populations (45) or methodologies employed, highlighting the necessity for further investigation to elucidate the underlying mechanisms and reconcile differences in findings across diverse clinical settings.

The present study identified statistically significant associations between serum calcium and several metabolic parameters, including asprosin, HDL-C, uric acid, and creatinine. Importantly, the association between serum calcium and asprosin remained significant in multivariable regression analysis, supporting its independence from major confounding factors. Although the magnitude of the correlation coefficients was modest, with asprosin explaining approximately 1.5% of the variance in serum calcium levels, such effect sizes are not unexpected in complex metabolic networks where multiple regulatory pathways coexist. Therefore, the observed associations should be interpreted as quantitatively modest but statistically robust. These findings may reflect subtle yet biologically meaningful interactions between calcium homeostasis and adipokine regulation, warranting further mechanistic and longitudinal investigation.

This study has several limitations. First, the cross-sectional design limits our ability to establish causality, as it can only confirm the correlation between serum calcium and asprosin, without determining the causal direction. Second, although common confounding factors such as age, sex, and BMI were adjusted for in this study, several potential factors were not measured or accounted for, including dietary calcium intake, vitamin D and parathyroid hormone (PTH) levels, estimated glomerular filtration rate (eGFR), and medication use. Third, the study’s reliance on total serum calcium and total asprosin measurements, without assessing biologically active ionized calcium, may underestimate or misrepresent the true association between these variables.

## 5 Conclusions

Serum asprosin was independently associated with serum calcium levels in elderly patients with T2D, suggesting a potential link between calcium homeostasis and adipokine regulation. Further longitudinal and mechanistic studies may help elucidate the clinical and biological implications of this relationship.

## Data Availability

The raw data supporting the conclusions of this article will be made available by the authors, without undue reservation.
